# Piezocatalytic nitrate reduction to ammonia in seawater

**DOI:** 10.1093/nsr/nwaf508

**Published:** 2025-11-14

**Authors:** Zhijie Li, Chaoqi Zhang, Yamin Xi, Yingying Zou, Tong Bao, Yingxuan Zhou, Rong Deng, Chao Liu, Chengzhong Yu

**Affiliations:** School of Chemistry and Molecular Engineering, East China Normal University; Shanghai 200241, China; School of Chemistry and Molecular Engineering, East China Normal University; Shanghai 200241, China; School of Chemistry and Molecular Engineering, East China Normal University; Shanghai 200241, China; School of Chemistry and Molecular Engineering, East China Normal University; Shanghai 200241, China; School of Chemistry and Molecular Engineering, East China Normal University; Shanghai 200241, China; School of Chemistry and Molecular Engineering, East China Normal University; Shanghai 200241, China; School of Chemistry and Molecular Engineering, East China Normal University; Shanghai 200241, China; School of Chemistry and Molecular Engineering, East China Normal University; Shanghai 200241, China; State Key Laboratory of Petroleum Molecular and Process Engineering, SKLPMPE, East China Normal University, Shanghai 200062, China; Shanghai Frontiers Science Center of Molecule Intelligent Syntheses, School of Chemistry and Molecular Engineering, East China Normal University, Shanghai 200062, China; School of Chemistry and Molecular Engineering, East China Normal University; Shanghai 200241, China; State Key Laboratory of Petroleum Molecular and Process Engineering, SKLPMPE, East China Normal University, Shanghai 200062, China; Australian Institute for Bioengineering and Nanotechnology, The University of Queensland, Brisbane 4072, Australia

**Keywords:** piezocatalysis, seawater, nitrate reduction, NH_4_^+^, metal phosphorous chalcogenides

## Abstract

The renewable-energy-driven conversion of nitrate in seawater into ammonia ions (NH_4_^+^) is a promising strategy for concurrent marine environmental remediation and sustainable chemical production. Herein, a seawater piezocatalytic nitrate reduction reaction (NitRR) is demonstrated for efficient NH_4_^+^ production by employing MnPS_3_ nanosheets (NSs) as a new type of piezocatalyst. It is shown that the Mn and P dual sites promote the adsorption and activation of NO_3_^−^ ions via an asymmetric side-on mode. Moreover, the lattice strain of MnPS_3_ NSs induced by the external mechanical vibration further weakens the N–O bond. With the additional contribution of the active hydrogen supplied by the S sites, the MnPS_3_ NSs exhibit an NH_4_^+^ production rate of 2.75 mmol h^−1^ g^−1^ in simulated seawater without sacrificial agents. By utilizing the mechanical energy of water flow in real seawater, the continuous conversion of NO_3_^−^ into NH_4_^+^ at a rate of 95.0% and with a selectivity of 96.4% is also achieved within 120 min. Our work paves the way for the sustainable conversion of nitrate in seawater into value-added chemicals via renewable natural energy.

## Teaser

In situformed hydrogen-bonded, robust yet deformable nanodomains effectively reinforce reversibly cross-linked networks, yielding super-strong, ultra-tough plastics with excellent thermal resistance, chemical stability, and reprocessability.

## INTRODUCTION

Seawater is one of the most abundant natural resources that accounts for >96% of Earth’s water [1]. Due to the ever-increasing population, the need for more food and the scarce freshwater on Earth, marine aquaculture is a fast-growing pillar sector that contributes to food security, sustainability and economic development [2]. Nevertheless, the frequent use of nutrients and fertilizers in farming as well as poorly managed sewage and wastewater discharge have caused seawater pollution [3, 4]. As a typical pollutant, the concentration of nitrate (NO_3_^−^) can reach ≤300 mg-N L⁻^1^ [5], causing negative ecological impacts such as eutrophication, reduced biodiversity and significant economic loss [3, 4]. While there is no universal standard for nitrate concentration regulation in seawater, a 30-day average concentration of 3.7? mg-N L^−1^ is recommended to protect marine aquatic life [6].

The prevailing strategies for NO₃⁻ contamination remediation in mariculture include physicochemical and biological approaches. The physicochemical method using porous materials [7] or membrane separation [8] is easy to operate, but the selectivity and overall performance need to be improved due to the added cost of treating concentrated by-products. The biological conversion of NO₃⁻ into N₂ (denitrification) represents an environmentally friendly approach, yet its marine application is challenged by high salinity and pretreatment steps [9]. To mitigate pollution due to NO_3_⁻ and use it as a valuable resource [10], the recently emerged nitrate reduction reaction (NitRR) to produce ammonia ions (NH_4_^+^) via photocatalysis or electrocatalysis in freshwater provides inspiration [4, 11]. The NitRR approach is expected to directly convert the NO₃⁻ in mariculture wastewater into NH_4_^+^ for crop fertilization (50–200 mg-N L⁻^1^) [12] without further extraction [13]. However, the NitRR in seawater has not been reported to the best of our knowledge.

In contrast to photocatalysis or electrocatalysis, piezocatalysis is capable of directly harnessing the environmental mechanical energy (e.g. water flow, sound, wind and vibration) to drive catalytic reactions without the need for light or electric energy [14, 15]. When piezocatalysts with non-centrosymmetric crystal structures are subjected to external mechanical vibrations, the misalignment of positive and negative charge centres results in a polarization electric field, promoting the generation of polarized charges for redox reactions [16]. Plenty of piezoelectric materials such as BaTiO_3_ [17] and CdS [18] have been investigated for applications such as water splitting, CO_2_ conversion and N_2_ reduction reactions [17–19], but not yet for the NitRR to produce NH_4_^+^ in either fresh water or seawater. We hypothesize that the ocean environment with inexhaustible mechanical wave energy [19] is ideal for driving the piezocatalytic NitRR process; nevertheless, the design of a high-performance piezocatalyst is indispensable.

**Scheme 1.**
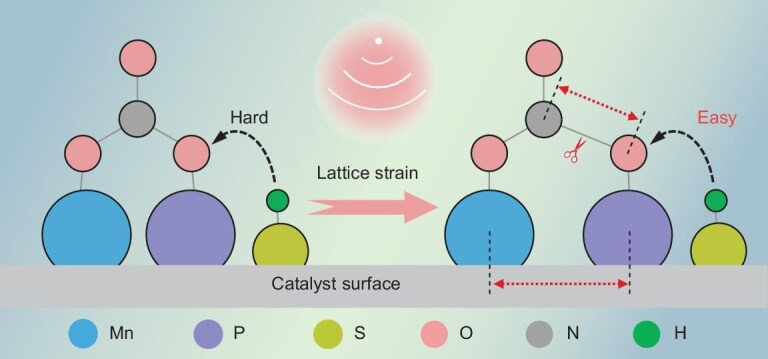
The working principle of piezocatalytic NitRR on MnPS_3_ NSs.

Herein, we demonstrate that MnPS_3_ nanosheets (NSs) are a novel piezocatalyst that achieves a piezocatalytic NitRR for efficient NH_4_^+^ production in seawater (Scheme 1). MnPS_3_ is a member of the metal phosphorous chalcogenides (MPCh_3_) family, with a typical 2D asymmetric crystalline structure [20, 21] and well-known photocatalytic or electrocatalytic functions [20, 22, 23]; however, its application in either piezocatalysis or the NitRR has not been reported. Systematic investigations reveal that the Mn and P dual sites absorb the NO_3_^−^ ions via an asymmetric side-on mode, inducing the polarization of NO_3_^−^. In addition, the external mechanical vibration leads to lattice strain in the MnPS_3_, further stretching and weakening the N–O bond. In combination with the concurrently produced polarized charges and the active hydrogen supplied by the S sites, the reduction and hydrogenation of NO_3_^−^ to NH_4_^+^ is promoted with a reduced energy barrier. As a result, the MnPS_3_ NSs exhibit an excellent piezocatalytic NitRR performance with an NH_4_^+^ production rate of 2.75 mmol h^−1^ g^−1^ in simulated seawater without sacrificial agents, which is superior to those of most reported photocatalytic NitRR and piezocatalytic nitrogen reduction reaction (NRR) systems. Moreover, the piezocatalytic NitRR can be performed by utilizing the mechanical energy of the water flow in real seawater for scalable NH_4_^+^ production at a NO_3_^−^ conversion rate of 95.0% and NH_4_^+^ selectivity of 96.4% within 120 min. This study has paved a new way for both nitrate remediation and NH_4_^+^ production for mariculture applications.

## RESULTS AND DISCUSSION

### Characterization of MnPS_3_

**Figure 1.**
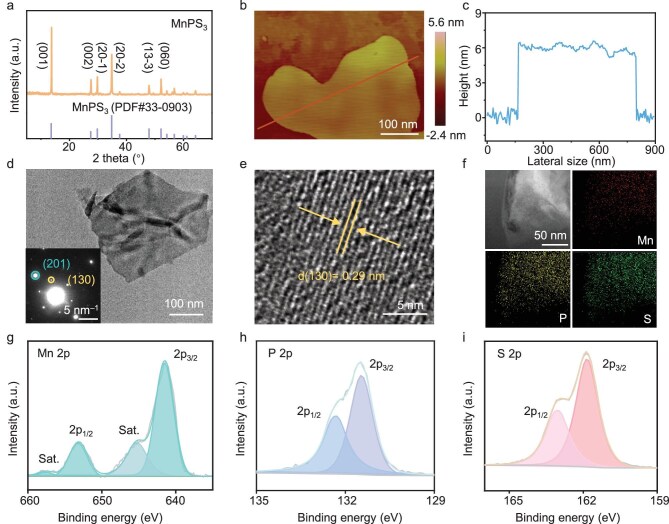
(a) XRD patterns of MnPS_3_ NSs. (b) Atomic force microscopy image. (c) Height distribution profile. (d) TEM image (inset: selected area electron diffraction pattern). (e) High-resolution transmission electron microscopy image. (f) High-angle annular dark-field scanning transmission electron microscopy and energy dispersive spectrometry elemental mapping images. High-resolution X-ray photoelectron spectroscopy spectra of (g) Mn 2p, (h) P 2p and (i) S 2p of MnPS_3_ NSs. Scale bars: (b, d) 100 nm, (e) 5 nm, (f) 50 nm.

Bulk MnPS_3_ was prepared by using a chemical vapour transport method [23]. The X-ray diffraction (XRD) pattern of the as-prepared sample (Fig. 1a) presents diffraction peaks at 13.6°, 27.5°, 29.75°, 34.8°, 47.9° and 52.1°, assigned to the (001), (002), (20$\bar{1}$), (20$\bar{2}$), (13$\bar{3}$) and (060) planes of MnPS_3_ (PDF #33–0903), respectively. The subsequent exfoliation treatment produced MnPS_3_ NSs. Compared with bulk MnPS_3_, the peak positions of MnPS_3_ NSs are almost unchanged. The peak intensity of the (001) plane against the (20$\bar{2}$) plane for MnPS_3_ NSs is increased compared with that of the bulk MnPS_3_ sample, possibly attributed to the nanosheet morphology with a more preferred orientation along the [001] direction than that of the bulk MnPS_3_, consistently with literature results [24].

The morphology and structure of MnPS_3_ NSs were investigated by using atomic force microscopy and transmission electron microscopy (TEM). As displayed in Fig. 1b, c and Fig. S1, MnPS_3_ NSs exhibit an ultra-thin nanosheet morphology with an average thickness of ∼6.2 nm. The TEM image (Fig. 1d) of the MnPS_3_ NSs also displays the nanosheet structure. The selected area electron diffraction pattern (inset in Fig. 1d) can be indexed to the [001] zone-axis of MnPS_3_ with a monoclinic phase, where spots of the {201} and {101} planes are observed. In the high-resolution TEM image (Fig. 1e), the clear lattice fringes with a spacing distance of 0.30 nm are attributed to the {130} plane of MnPS_3_. The high-angle annular dark-field scanning TEM and corresponding energy dispersive spectrometry elemental mapping images (Fig. 1f) show the even distribution of Mn, P and S elements in MnPS_3_ NSs. The Mn/P/S molar ratio is determined to be ∼1:1:3, which is close to the composition of stoichiometric MnPS_3_.

X-ray photoelectron spectroscopy (XPS) was further applied to explore the electronic structure of MnPS_3_ NSs. In the XPS survey spectrum (Fig. S2), the Mn, P and S elements were detected at a Mn/P/S atomic ratio of 1:0.98:2.97, in accordance with the results of element analysis by using TEM. The Mn 2p spectrum (Fig. 1g) shows two peaks of Mn 2p_3/2_ and 2p_1/2_ orbits at 641.6 and 653.2 eV with two corresponding satellite peaks at 645.4 and 657.7 eV, respectively. The P 2p spectrum (Fig. 1h) can be divided into two peaks at 131.5 and 132.4 eV, assigned to P 2p_3/2_ and P 2p_1/2_, respectively. For S 2p (Fig. 1i), the peaks located at 161.8 and 163.0 eV are attributed to S 2p_3/2_ and S 2p_1/2_, respectively. These results collectively demonstrate the successful fabrication of ultra-thin MnPS_3_ nanosheets.

**Figure 2.**
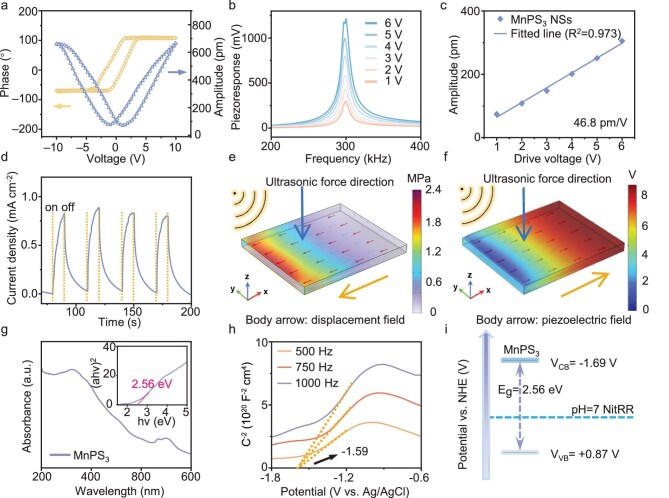
(a) Butterfly-shaped amplitude curve and phase hysteresis loop. (b) PFM resonance peaks under different voltages. (c) Effective piezoelectric coefficient. (d) Transient piezo-current response. Finite element simulations of (e) bulk strain and displacement field distribution and (f) corresponding bulk piezo potential and polarization direction distribution of MnPS_3_ under ultrasonic pressure. (g) UV–vis DRS and corresponding Tauc plot. (h) Mott–Schottky plots at different frequencies. (i) Band structure of MnPS_3_.

The piezoelectric property of MnPS_3_ NSs was investigated by using piezoelectric force microscopy (PFM). As shown in Fig. S3, the PFM amplitude and phase mapping signals reveal the nanosheet morphology and the generation of a uniform piezoelectric response within the nanosheets [15]. The switching phase and amplitude loops were further recorded by using an in-plane resonance-enhanced PFM mode under an alternating-current (AC) electric field (Fig. 2a). The phase loop exhibits a well-defined 180° phase-reversal hysteresis. The voltage-dependent amplitude loop shows a typical butterfly-like shape, suggesting a switchable polarization feature of MnPS_3_ NSs [25]. Figure 2b displays the piezoresponse curves under AC driving voltages from 1 to 6 V. The intensity of the piezoresponse peak at a resonance frequency of ∼300 kHz increases as the driving voltage rises. After correction by using the quality factor derived from the damped harmonic oscillator model [26], the piezoresponse signal exhibits a linear dependence on the driving voltage, indicative of a piezoresponse feature of MnPS_3_ NSs [27]. The relatively lower resonance frequency compared with those of conventional piezoelectric materials (e.g. lead zirconate titanate, 380 kHz) indicate that MnPS_3_ NSs are more sensitive to applied mechanical force [28]. The piezoelectric coefficient of MnPS_3_ NSs was estimated to be 46.8 pm/V (Fig. 2c), which is higher than those of many reported piezocatalysts [29].

Furthermore, the transient piezo-current response profile of MnPS_3_ NSs under ultrasonic on/off switching presents obvious piezo-current signals (Fig. 2d), indicating the formation of polarized charges over MnPS_3_ with mechanical energy input. To understand the mechanical-force-induced piezoelectric response, finite element method analysis was performed by using the COMSOL multi-physic software. When forces are applied in the in-plane direction, the MnPS_3_ nanosheet is deformed, with the generation of a displacement field and a corresponding piezoelectric field (Fig. 2e and f), promoting the piezo-electron transfer for driving piezocatalysis [17]. Even with a centrosymmetric C2/m structure, the interlayer sliding in ultra-thin MnPS_3_ nanosheets (Fig. S4) may reduce the structural symmetry, thus inducing the piezoelectric response. A similar phenomenon has been widely observed in 2D material-based piezoelectric catalysts with symmetric crystalline structures [30–33].

Based on the energy-band theory, the piezoelectricity-generated electrons and holes migrate to the conduction band (CB) and valance band to drive reduction and oxidation reactions, respectively [14]. To further investigate the charge separation induced by the piezoelectricity, scanning Kelvin probe force microscopy (KPFM) measurements were performed. The KPFM potential maps and corresponding potential distribution profiles of pristine MnPS_3_ NSs and MnPS_3_ NSs under mechanical stress are displayed in Fig. S5a–d. In comparison with those of pristine MnPS_3_ NSs, the surface potential of the mechanically stressed sample decreases by 17 mV, suggesting the migration of piezoelectricity-generated electrons to the surface of the MnPS_3_ NSs to participate in the reduction reaction. To determine the band structure of the MnPS_3_ NSs, Ultravioletvisible (UV-vis) diffuse reflectance spectroscopy (DRS) and Mott–Schottky measurements were performed. Based on the UV–vis spectrum and corresponding Tauc plot (Fig. 2g), the bandgap (*E*_g_) of MnPS_3_ NSs is estimated to be 2.56 eV, in accordance with the literature result [34]. Figure 2h presents the Mott–Schottky curves for MnPS_3_ NSs at different frequencies, implying an n-type semiconductor characteristic. The flat-band potential (*E*_fb_) of MnPS_3_ NSs is determined to be –1.59 V versus that of a normal hydrogen electrode (NHE). For n-type semiconductors, the bottom of CB is generally ∼0.1 V lower than the *E*_fb_, thus the CB value is calculated to be –1.69 V. Considering further the *E*_g_ value, the band structure of MnPS_3_ NSs is depicted in Fig. 2i, showing that the piezocatalytic nitrate reduction to NH_4_^+^ (–0.12 V vs. NHE, pH = 7) is thermodynamically favourable over MnPS_3_ NSs. During piezocatalysis, the band structure of the piezocatalyst can be modulated by the vibration-induced stress that triggers piezoelectric polarization and charge accumulation [14]. Therefore, the band structures of pristine and stressed MnPS_3_ were firstly studied by using density functional theory (DFT) calculation. A lattice-stretched model (*a* = 12.15 Å, *b* = 25.31 Å, *c* = 21.46 Å) was established by applying 100 MPa of pressure to the original MnPS_3_ NSs (*a* = 12.15 Å, *b* = 21.30 Å, *c* = 21.46 Å). The structural stretch along the *b*-axis results in an elongation of the Mn–P bonds from 3.63 to 3.81 Å (Fig. S6). The band gap of pristine MnPS_3_ was calculated to be 2.50 eV (Fig. S7a)—close to the experimental result. Compared with that of pristine MnPS_3_, the band gap of the stressed sample is narrowed by 0.57 eV (Fig. S7b), which may facilitate the generation of electron–hole pairs and promote the charge transfer during the piezocatalytic NitRR [15].

### Piezocatalytic NitRR performance in simulated seawater

**Figure 3.**
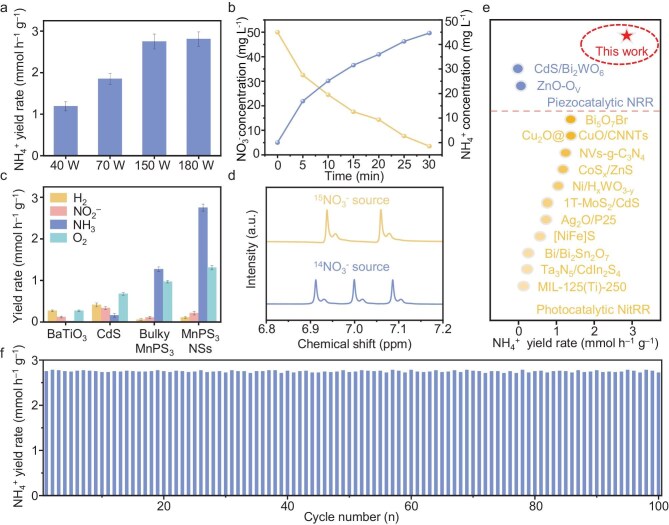
Piezocatalytic NitRR performance of MnPS_3_ nanosheets. (a) NH_4_^+^ yields of MnPS_3_ NSs under various ultrasonic powers. (b) Time-dependent concentration evolution curves of NO_3_^−^ and NH_4_^+^. (c) NH_4_^+^ and by-product yields of MnPS_3_ NSs, bulky MnPS_3_, CdS and BaTiO_3_ at 150 W. (d) ^1^H NMR spectra of NH_4_^+^ produced by using ^14^NO_3_^−^ and ^15^NO_3_^−^ as the raw materials. (e) Comparison of NH_4_^+^ yield of MnPS_3_ with reported photocatalytic NitRR and piezocatalytic NRR systems. (f) Recycling performance of MnPS_3_ under 40 kHz of ultrasonic vibration.

The piezocatalytic NitRR performance of MnPS_3_ NSs for NH_4_^+^ production was evaluated firstly in simulated seawater containing 0.5 M of NaCl and 100 mg L^−1^ of NO_3_^−^ in the absence of light and sacrificial agents under ultrasonication. The NH_4_^+^ concentration was determined by using ^1^H nuclear magnetic resonance (NMR) spectroscopy. As shown in Fig. 3a, the NH_4_^+^ production rate of the MnPS_3_ NSs rapidly increased from 1.19 to 1.85 to 2.75 mmol h^−1^ g^−1^ with ultrasonic power that elevated from 40 to 70 to 150 W. However, only a slight improvement in the NH_4_^+^ yield to 2.81 mmol h^−1^ g^−1^ was observed under 180 W, suggesting that a further increase in ultrasonic power could not enhance the piezocatalytic NitRR activity. Therefore, 150 W was chosen as the optimal ultrasonic power in the following experiments.

**Figure 4.**
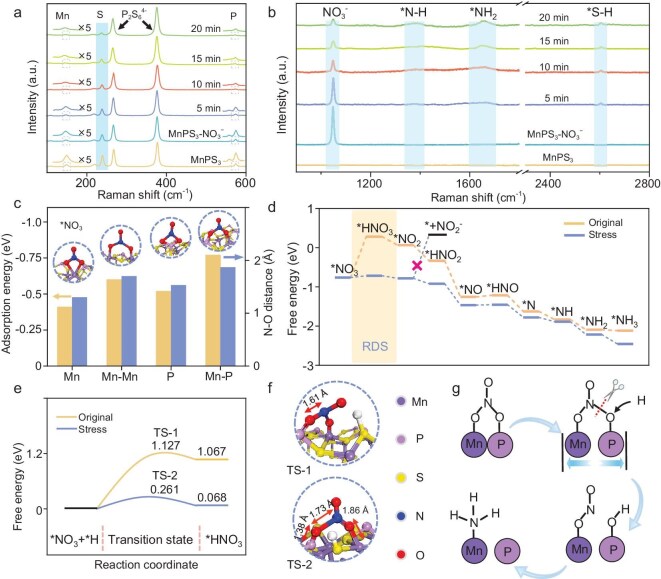
(a, b) Raman spectra of MnPS_3_ suspension without NO_3_^−^ and reaction solution containing MnPS_3_ and NO_3_^−^ without sonication and with sonication for 5, 10, 15 and 20 min. (c) Adsorption energies and N–O distances of four adsorption configurations on MnPS_3_ NSs. (d) Gibbs free-energy profiles of piezocatalytic NitRR. (e) Free-energy diagram of *NO_3_ hydrogenation to *HNO_3_ on MnPS_3_ NSs and stretched MnPS_3_ NSs. (f) Adsorption configurations and N–O distances of TS-1 and TS-2. (g) Scheme of lattice-strain-promoted NO_3_^−^ activation on stretched MnPS_3_ NSs.

Figure 4b displays the time-dependent concentration change in NO_3_^−^ and NH_4_^+^ on MnPS_3_ NSs. As the reaction proceeded, the NO_3_^−^ is gradually consumed with the continuous accumulation of NH_4_^+^, ultimately reaching a NO_3_^−^ conversion efficiency of 99.6% and an NH_4_^+^ selectivity of 96.3% at 3 min. For comparison, bulky MnPS_3_ and two widely investigated piezocatalysts including BaTiO_3_ and CdS were also synthesized and tested (Fig. S8). For bulky MnPS_3_, BaTiO_3_ and CdS, the NH_4_^+^ production rates were measured to be 1.27, 0.02 and 0.16 mmol h^−1^ g^−1^ under 150 W, respectively—all inferior to that of MnPS_3_ NSs (Fig. 3d and Fig. S9). Among the three samples, MnPS_3_ NSs show the lowest yield rates of by-products including H_2_ (0.11 mmol h^−1^ g^−1^) and NO_2_^−^ (0.21 mmol h^−1^ g^−1^). In addition, the production rate of O_2_ (the product of the coupled water oxidation reaction) for MnPS_3_ NSs was determined to be 1.31 mmol g^−1^ h^−1^, also exceeding those of bulky MnPS_3_ (0.97 mmol g^−1^ h^−1^), BaTiO_3_ (0.27 mmol g^−1^ h^−1^) and CdS (0.68 mmol g^−1^ h^−1^). Therefore, the overall NitRR to ammonia follows the equation of NO_3_^−^ + 3H_2_O → NH_4_^+^ + 2O_2_ + 2OH^−^. Notably, the NH_4_^+^ production performance of MnPS_3_ NSs outperforms those of most of the reported photocatalysts in photocatalytic NitRR and piezocatalytic NRR systems (Fig. 3e) [35–47]. Furthermore, the ability to produce NH_4_^+^ has also been evaluated under stirring conditions to simulate seawater fluctuations. As the stirring speed increases from 100 to 1000 r/min, the NH_4_^+^ yield rate is elevated from 0.54 to 2.17 mmol h⁻¹ g_cat_⁻¹ (Fig. S10).

To verify the N source of the produced NH_4_^+^, an isotopic-labelling experiment was carried out. As displayed in the NMR spectra (Fig. 3d), triplet ^14^NH_4_^+^ and doublet ^15^NH_4_^+^ signals were detected by using ^14^NO_3_^−^ and ^15^NO_3_^−^ as the feedstocks, respectively (Figs S11 and S12). The ^15^ NH_4_^+^ yield of MnPS_3_ NSs is measured to be 2.71 mmol h^−1^ g^−1^, which is close to the value for ^14^ NH_4_^+^. Besides, the parallel control experiments suggest that almost no NH_4_^+^ was detected in the reaction systems without piezocatalyst, ultrasonication or nitrate during the piezocatalytic reaction (Fig. S13). These results collectively validate that the produced NH_4_^+^ stemmed from piezocatalytic NO_3_^−^ conversion over MnPS_3_ NSs.

Apart from the activity, the piezocatalytic stability of MnPS_3_ NSs was also studied. After 100 consecutive cycles in a total time window of 100 h, a negligible drop in the NH_4_^+^ production rate was found, revealing the robust piezocatalytic stability of MnPS_3_ NSs (Fig. 3f). As revealed by TEM, XRD and XPS tests, the MnPS_3_ NSs after the tests exhibit well-preserved morphology, crystalline structure and chemical composition, demonstrating excellent structural stability during the piezocatalytic NitRR process (Figs S14–S16 and Table S2). The absence of Cl^−^ signals in the XPS spectra of used MnPS_3_ suggests an excellent Cl^−^-repelling property in seawater, consistently with our previous findings [24].

### Piezocatalytic mechanism investigation

*In situ* Raman spectroscopy was applied to monitor the piezocatalytic NitRR over MnPS_3_ NSs. The Raman spectra of the MnPS_3_ suspension without NO_3_^−^ and reaction solutions containing MnPS_3_ and NO_3_^−^ without sonication and with sonication for 5 and 10 min are shown in Fig. 4a and b. In the spectrum for the MnPS_3_ suspension, the peaks at 147.5, 238.5 and 572.4 cm^−1^ belong to the in-plane optical mode of Mn (E^2^_g_), out-of-plane vibration of S (A^1^_1g_) and the stretching vibration of P–P (A^3^_1g_), respectively [48]. Compared with the spectrum for the MnPS_3_ suspension, a strong peak of the NO_3_^−^ stretching mode is observed at 1050 cm^−1^, with the peak intensities of Mn (E^2^_g_) and P–P (A^3^_1g_) decreased in the spectrum of the MnPS_3_ after soaking in KNO_3_ solution without sonication, suggesting the lowered charge density of the Mn and P sites after the adsorption of electron-withdrawing NO_3_^−^. After sonication treatment for 5 min, the peaks assigned to the stretching vibration of N–H and δ_a_(HNH) of NH_4_^+^ (1381 and 1651 cm^−1^) are generated with the peak intensities of the NO_3_^−^ reduced. Besides, another new peak of the stretching vibration of the S–H_ads_ bond emerged at 2599 cm^−1^, with the attenuation of the S (A^1^_1g_) peak, which may have resulted from the combination of S^2−^ with the H in the H_2_O. By prolonging the reaction time to 10 and 20 min, the peaks of E^2^_g_, A^1^_1g_, A^3^_1_  _g_ and NO_3_^−^ are further weakened with intensified *N–H and *NH_2_ peaks and an almost unchanged S–H peak. The *in situ* Raman results suggest that the Mn and P sites adsorb NO_3_^−^ and the S sites capture active hydrogen from the H_2_O, collaboratively promoting the hydrogenation of NO_3_^−^ to NH_4_^+^ over MnPS_3_.

The XPS spectrum of MnPS_3_ after soaking in 0.1 M of KNO_3_ solution (the collected sample was denoted as MnPS_3_–NO_3_^−^) for 10 min was also recorded, showing the existence of Mn, P, S, N and O elements. The N 1s spectrum of MnPS_3_–NO_3_^−^ (Fig. S17a) exhibits the characteristic peaks of N–O and π–π* in NO_3_^−^ at 399.1 and 406.3 eV, respectively. Compared with the Mn 2p spectrum of MnPS_3_ (Fig. S17b), two additional peaks attributed to the 2p_3/2_ and 2p_1/2_ orbitals of the Mn–O bond emerge at 644.4 and 656.2 eV for MnPS_3_–NO_3_^−^, respectively, which may have stemmed from the chemical adsorption of NO_3_^−^ on the Mn sites. Besides, the binding energies of Mn 2p_3/2_ and Mn 2p_1/2_ for MnPS_3_–NO_3_^−^ positively shift by 1.1 and 1.3 eV, respectively, due to the electron-withdrawing effect of NO_3_^−^ [49]. For the P 2p spectrum of MnPS_3_–NO_3_^−^ (Fig. S17c), the binding energies of the original P 2p_3/2_ and P 2p_1/2_ peaks are also higher than those for MnPS_3_, with the generation of a new peak assigned to the P–O bond. The XPS observations further suggest that both Mn and P act as the NO_3_^−^ adsorption sites, consistently with the results of the Raman spectra. Figure S18 presents the diffuse reflectance infrared Fourier transform spectrum (DRIFTS) of MnPS_3_ after soaking in 0.1 M of KNO_3_ solution (MnPS_3_–NO_3_^−^), with pure MnPS_3_ and KNO_3_ solid as a comparison. The spectrum of KNO_3_ shows the typical peak of NO_3_^−^ at 1352 cm^−1^, whereas no distinct peak is detected in the spectrum of pure MnPS_3_ near this region. For MnPS_3_–NO_3_^−^, two additional peaks attributed to the side-on NO_3_^−^ adsorption mode were observed at 1370 and 1432 cm^−1^, in accordance with the literature results [50]. Combining the Raman, XPS and DRIFTS results together, it is speculated that NO_3_^−^ is adsorbed on Mn–P dual sites via an asymmetric side-on mode.

To gain further insight into the piezocatalytic NitRR process over MnPS_3_ NSs, DFT calculations were carried out. First, the adsorption configurations and energies of NO_3_^−^ on MnPS_3_ NSs were calculated. Four possible NO_3_^−^ adsorption configurations (Fig. 4c) were considered, including end-on adsorption on Mn and P single sites and side-on adsorption on Mn–Mn and Mn–P dual sites. Given the long distance between two adjacent P sites (6.08 Å), the possibility of side-on adsorption on P–P dual sites is excluded. As shown in Fig. 4c, the adsorption energy of the asymmetric side-on configuration on Mn–P dual sites is the lowest (–0.77 eV) among the four configurations, indicating that NO_3_^−^ adsorption is thermodynamically more favourable on Mn–P dual sites. Furthermore, the N–O bond lengths of NO_3_^−^ adsorbed on single Mn and P sites are 1.30 and 1.70 Å, respectively. When adsorbed on Mn–P dual sites, the N–O bond is stretched to 1.87 Å, which is also longer than that of NO_3_^−^ adsorbed on Mn–Mn dual sites (1.53 Å). The DFT results indicate that the asymmetric side-on adsorption mode of NO_3_^−^ on Mn–P dual sites weakens the N–O bond, which is beneficial for the piezocatalytic NitRR.

Figure 4d and Fig. S19 present the free-energy change (Δ*G*) in each step during the piezocatalytic NitRR process and the corresponding intermediate configurations. Generally, the piezoelectric effect originates from the piezo potential in response to deformation of the piezoelectric materials under mechanical force (e.g. ultrasonication). The Δ*G* values of all the reduction/hydrogenation steps on stretched MnPS_3_ are lower than those on pristine MnPS_3_. Among the complex nine-proton coupled eight-electron process of the NitRR, the first hydrogenation step of *NO_3_→*HNO_3_ is recognized as the rate-determining step (RDS). Δ*G* of the RDS for stretched MnPS_3_ is −0.72 eV, which is 0.98 eV lower than that of pristine MnPS_3_ (0.26 eV, thermodynamically unfavourable), indicating that the stretched state of MnPS_3_ reduces the energy barrier significantly for facilitating NO_3_^−^ hydrogenation. The higher energy barrier of *NO₂ desorption to its further hydrogenation inhibits the yield of NO_2_^−^ by-products. Moreover, the high energy cost from H* intermediate to H_2_ (0.68 eV, Fig. S20) can impede the competitive hydrogen evolution reaction (HER) and facilitate the hydrogenation of NO_3_^−^ to NH_3_, in accordance with the experimental results.

To further understand the role of the lattice strain in promoting the NitRR, the reaction kinetics of *NO_3_→*HNO_3_ was investigated (Fig. 4e). The energy cost from adsorption *NO_3_ to transition state (TS-1) for pristine MnPS_3_ (1.127 eV) is higher than that of TS-2 for stretched MnPS_3_ (0.261 eV), implying that the hydrogenation of *NO_3_ is kinetically more favoured. The optimized structure of TS-2 (Fig. 4f) shows that the P–O bond (1.38 Å) is shorter than the Mn–O bond (1.86 Å), with the elongation of the N–O bond at the P side, suggesting a stronger adsorption of NO_3_^−^ on the P site than that on the Mn site. Consequently, for *NO_3_ hydrogenation, the O bonded on the P site is easier to be attacked by the active hydrogen generated at the Lewis basic S sites through H_2_O dissociation. Moreover, the N–O bond length of NO_3_^−^ at the P side on pristine MnPS_3_ is 1.61 Å, which is shorter than that for stretched MnPS_3_ (1.73 Å). The results indicate that the relatively larger Mn–P distance in the stretched MnPS_3_ (from 3.63 to 3.81 Å) is conducive to weakening the N–O bond of NO_3_^−^, lowering the energy barrier for the hydrogenation of *NO_3_ to *HNO_3_ (Fig. 4f).

### Scale-up system in real seawater

**Figure 5.**
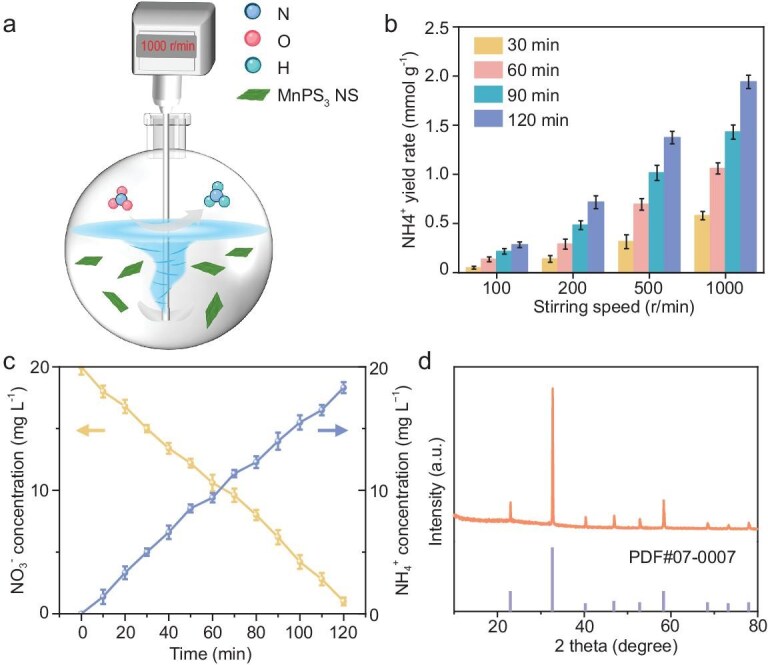
(a) Schematic illustration of piezocatalytic NitRR under mechanical stirring. (b) NH_4_^+^ yield rate under different stirring speeds. (c) Concentration of NO_3_^−^ and NH_4_^+^ versus time under 1000 r/min. (d) XRD pattern of solid NH_4_Cl.

To further explore the application potential of the piezocatalytic NitRR over MnPS_3_, a scale-up test was conducted in 2 L of real seawater sampled from a marine aquaculture farm with a NO_3_^−^ concentration of 20 mg L^−1^ (Fig. 5a and Fig. S21). The piezocatalytic NitRR was driven by the mechanical energy generated from the water flow under stirring conditions. With the stirring speed increasing from 100 to 1000 r/min, the NH_4_^+^ yield rate is elevated from 0.35 to 1.76 mmol h^−1^ g_cat_^−1^ (Fig. 5b), showing water-flow strength-dependent behaviour. The time-dependent piezocatalytic NitRR profile under 1000 r/min reveals the continuous conversion of NO_3_^−^ into NH_4_^+^ at a NO_3_^−^ conversion rate of 95.0% and NH_4_^+^ selectivity of 96.4% within 120 min (Fig. 5c). Afterwards, the produced NH_3_ was purged with air, absorbed in hydrochloric acid solution and then converted into solid NH_4_Cl (an agricultural fertilizer) through rotary evaporation. The XRD pattern of the resulting powder (Fig. 5d) matches well with those of standard NH_4_Cl crystals (PDF#07–0007). To further enhance the practical application potential, a solar-assisted piezocatalytic NitRR system was established (Fig. S22). In combination with the solar-driven photocatalysis process, the NH_4_^+^ yield rate increases to 3.69 mmol h⁻¹ g_cat_⁻¹ (Fig. S23), which is 1.34-fold higher than that under ultrasound-only conditions.

## CONCLUSION

In summary, it is demonstrated that MnPS_3_ NSs are an effective and robust piezocatalyst that realizes piezocatalytic NitRR with a NH_4_^+^ production rate of 2.75 mmol h^−1^ g^−1^ in seawater. The active sites of MnPS_3_ NSs and the piezocatalytic NitRR are also revealed. The Mn and P dual sites adsorb the NO_3_^−^ ions via an asymmetric side-on mode, enhancing the polarization and activation of NO_3_^−^. The N–O bond is further weakened by the lattice strain of MnPS_3_ NSs induced by external mechanical vibration. With the active hydrogen supplied by S sites in the MnPS_3_ NSs, efficient piezocatalytic NitRR performance is achieved. The piezocatalytic NitRR system also allows the scalable production of NH_4_^+^ by utilizing the mechanical energy of the water flow in real seawater, opening up a new paradigm for marine resource valorization.

## METHODS

The details about the sample synthesis and characterization are included in the online Supplementary data.

## Supplementary Material

nwaf508_Supplemental_File
