# Supplementary material for: Piezocatalytic nitrate reduction to ammonia in seawater
Source: Natl Sci Rev. 2025 Nov 14;13(1):nwaf508. doi: 10.1093/nsr/nwaf508 (PMC12805826; doi:10.1093/nsr/nwaf508)
Supplement: nwaf508_Supplemental_File [file nwaf508_supplemental_file.pdf]

Supplementary Data

Piezocatalytic Nitrate Reduction to Ammonia in Seawater

Zhijie Li,[1] Chaoqi Zhang,[1] Yamin Xi,[1] Yingying Zou,[1] Tong Bao,[1] Yingxuan Zhou,[1] Rong Deng,[1] Chao Liu, [1,2,3*] and Chengzhong Yu[1,2,4*]

[1]School of Chemistry and Molecular Engineering, East China Normal University; Shanghai 200241, China;

[2]State Key Laboratory of Petroleum Molecular and Process Engineering, SKLPMPE, East China Normal University, Shanghai 200062, China;

[3]Shanghai Frontiers Science Center of Molecule Intelligent Syntheses, School of Chemistry and Molecular Engineering, East China Normal University, Shanghai 200062, China;

[4]Australian Institute for Bioengineering and Nanotechnology, The University of Queensland, Brisbane 4072, Australia

***Corresponding authors**. E-mails: cliu@chem.ecnu.edu.cn; czyu@chem.ecnu.edu.cn; c.yu@uq.edu.au

Materials and Synthetic procedures

Materials

Red phosphorus (99.999%) and sulfur (99.999%) were obtained from STREM, Germany. Manganese (99.9%) powder (−100 mesh) was obtained from Alfa Aesar, Germany. Isopropanol (IPA, 99%) and acetone (99%) were purchased from Sigma-Aldrich. D_2O (99.9%, Macklin), maleic acid (99%, J&K) and ^{15}N-nitrate (99%, Shanghai Yuanye Co, Ltd) were used as received. Deionized water (18.2 MΩ·cm) was made from Purelab Ultra.

Materials Characterization

Transmission electron microscopy (TEM) images were collected on a JEM-2100F (JEOL, Japan) with an acceleration voltage of 200 kV. Scanning electron microscopy (SEM) images were acquired by a scanning electron microscope (HITACHI-S4800). Bruker Dimension Icon atomic force microscope (AFM) was used to characterize the thickness of materials. The piezoelectric feature and surface electric potential were explored by the Kelvin probe force microscopy (KPFM) and piezoresponse force microscopy (PFM) modules. Wide-angle X-ray diffraction (XRD) patterns were recorded on a Bruker D8 Advance Powder X-ray diffractometer (Bruker AXS, Germany) using Cu-Kα radiation source. X-ray photoelectron spectroscopy (XPS) measurement was carried out on a Thermo ESCALAB 250 using an Al Kα radiation and C 1s (284.8 eV) as a reference to correct the binding energy. Diffuse reflectance UV-vis absorption spectroscopy was recorded in the spectral region of 200-800 nm on a UV-vis spectrophotometer (Lambda950). Raman spectra of the samples were acquired on a GX-PT-1500 instrument with an incident wavelength of 633 nm. Gas chromatography (GC, Aglient Technologies 7890B) was used to detect the gas products. Nuclear Magnetic Resonance (NMR) Spectrometer (600 MHz ^1H NMR) was used to quantify the ammonia concentration.

Synthesis of metal phosphorous trichalcogenides

A mixture containing 0.549 g of metal, 0.309 g of phosphorus and 0.321 g of sulfur was sealed in a quartz glass ampule (30 × 150 mm; wall thickness 3 mm) with high vacuum (below 1×10^{-3} Pa). The ampule was then placed in the muffle furnace and heated at 650 °C for 120 h with a heating rate of 2 °C/min. After cooling down to room temperature, the resultant MPS_3 powder was collected for further use.

Preparation of $MnPS_3$ nanosheets

$MnPS_3$ nanosheets were fabricated by a liquid exfoliation method. In detail, 500 mg of bulk $MnPS_3$ powder was added to 50 mL of ethanol with ultrasonication in an ice bath for 12 h. Afterwards, the prepared dispersions were centrifuged at 9000 r/min for 3 min and the supernatant was reserved for further use.

Finite element method analysis

Finite element method (FEM) analysis was performed by using the COMSOL multiphysics software to simulate the induced displacement field and corresponding piezoelectric field on $MnPS_3$ nanosheets. The pressure was settled as 100 MPa to simulate the force from rupture of cavitation bubbles during the ultrasonic process [1,2]. The geometrical parameters of the length (L), width (W), and thickness (T) are set as 600 nm, 300 nm and 6 nm, respectively, corresponding to the PFM topography of $MnPS_3$.

Piezocatalytic performance measurement

In a typical process, 35 mg of $MnPS_3$ nanosheet powder was dispersed into a 100 mL borosilicate tube containing 50 mL simulated seawater with 0.5 M NaCl and 100 mg L^{-1} NO_3^-, which was continuously bubbled with Ar for 30 min to remove air before ultrasonic treatment. To initiate the piezocatalytic reaction, a constant temperature numerically controlled ultrasonic cleaner (KQ-600GVDV, 300 W, 45 kHz) was used to provide mechanical energy. The whole experiment was carried out in the dark and the reaction temperature was maintained at 25 °C (± 0.5 °C) by a cooling circulation system. 5 mL of the reaction solution was collected at given times and mixed with 5 mL of 0.1 M HCl, 50 μL of D_2O and 5 mL of maleic acid (50 ppm) for determining the $^{14}NH_4^+$ concentration by NMR. The ^{15}N isotope labeling experiment was performed by using high purity $^{15}NO_3^-$ as the nitrogen source with the same reaction procedure as mentioned above. The ammonia concentration was quantified by the standard calibration curves.

Piezo-photocatalytic NitRR performance measurement

The piezo-photocatalytic NitRR performance was evaluated under simultaneous ultrasonic vibration and solar light irradiation. Ultrasonic vibration was supplied by a cleaner (KQ-600GVDV, 300 W, 45 kHz), and simulated solar light was provided by a 300 W xenon lamp (PLS-SXE300D/300DUV, Beijing Perfectlight). In a typical process, 35 mg of $MnPS_3$ nanosheet powder was dispersed in 50 mL of simulated seawater (0.5 M NaCl and 100 mg L^{-1} NO_3^-) within a piezo-photocatalytic reactor. The mixture was purged with Ar for 30 minutes prior to the reaction to

remove dissolved oxygen. The reaction temperature was maintained at 25 °C (± 0.5 °C) by a cooling circulation system. At given time intervals, a certain volume of the supernatant was collected and filtrated with a millipore filter (0.22 μm) to separate the $MnPS_3$ NSs. The ammonia concentration was quantified by the standard calibration curves and NMR.

Piezocurrent response Test

Piezocurrent response measurements were performed on a Chenhua CHI 760E electrochemical workstation by a standard three-electrode cell system in 0.1 M KNO_3 electrolyte. The Pt wire, Ag/AgCl, and piezocatalyst modified indium-doped tin oxide (ITO) were used as counter, reference and working electrodes, respectively. The working electrode was prepared as follows: 10 mg of catalysts was first mixed with 30 μL of 10% Nafion solution to make a slurry. Afterward, the slurry was pipetted onto a piece of ITO glass (effective area: 1 cm^2) and then dried at 55 °C overnight. The piezocurrent was recorded under ultrasonic using a constant temperature numerically controlled ultrasonic cleaner (KQ-600GVDV, 300 W, 45 kHz) as ultrasonic source.

In situ Raman tests

In situ Raman spectra were detected on a LabRAM HR Evolution (Horiba) equipped with OLYMPUS BX41 Raman instrument. The sample cell was type-K004 (Tianjin Aida), equipped with a 10 mm × 10 mm silicon substrate. Before the test, the ink composed of 10 mg of piezocatalysts, 500 μl H_2O, 460 μl isopropanol and 40 μl Nafion was coated on the silicon substrat and dried naturally. The sample cell was fill with 0.1 M KNO_3 solution and purged with Ar for 30 min. The spectra were then collected under sonication using a 300 W ultrasonic vibration rod at 785 nm.

Computational Details

Spin-polarized density functional theory (DFT) calculations were performed using VASP package [3,4]. The exchange-correlation energy was treated based on the generalized gradient approximation (GGA) by using the Perdew-Burke-Ernzerhof (PBE) functional [5]. The projected augmented wave (PAW) potentials were chosen to describe the ionic cores and take valence electrons into account using a plane wave basis set with a kinetic energy cutoff of 500 eV. Partial occupancies of the Kohn-Sham orbitals were allowed using the Gaussian smearing method and a width of 0.05 eV. Geometries were optimized until the energy had converged to 10^{-5} eV and the force converged to 0.02 eV/Å. The structural models of the molecules were constructed based on their standard structural formula. The vacuum spacing in a direction perpendicular to the plane of the structure

was set at 15 Å. A 2×2×1 Monkhorst-Pack k-point mesh was used for sampling the Brillouin zone for the relaxation and self-consistency of calculations [6]. Finally, the adsorption energies (E_{ads}) were calculated as E_{ads}= $E_{ad/sub}$ -E_{ad} -E_{sub}, where $E_{ad/sub}$, E_{ad}, and E_{sub} are the total energies of the optimized adsorbate/substrate system, the adsorbate in the structure, and the clean substrate, respectively. The free energy was calculated using the equation:

$G = E + ZPE – TS$,

where G, E, ZPE and TS are the free energy and total energy from DFT molecular frequency calculations, zero-point energy and entropic contributions, respectively. In $MnPS_3$ nanosheets calculation, the top two layers were relaxed while the other layers fixed.

Results and Discussion

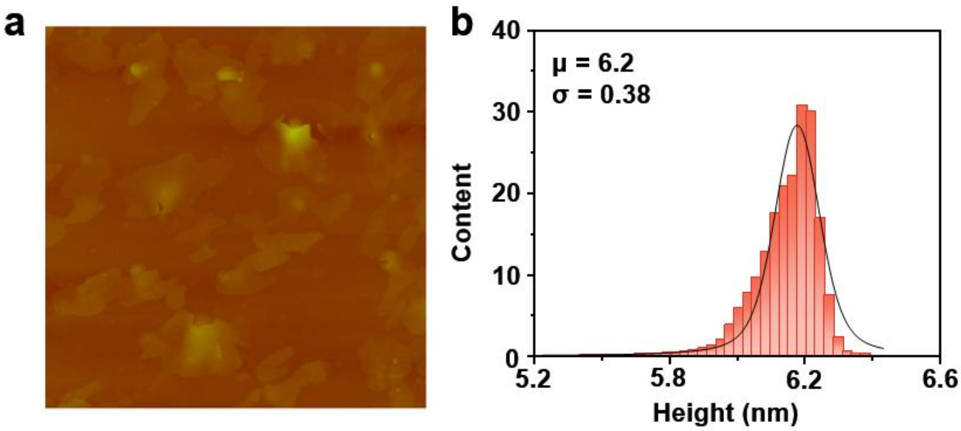

Figure S1. (a) AFM image and (b) statistical histograms of thickness of $MnPS_3$ nanosheets.

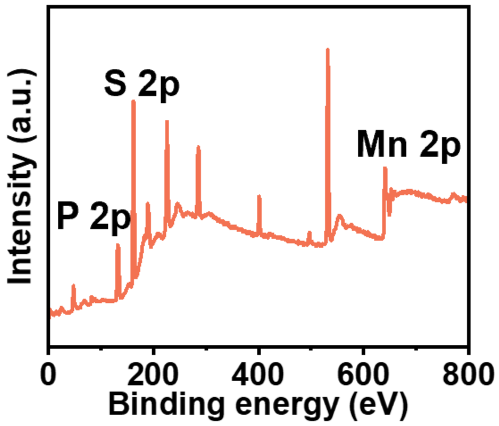

Figure S2. XPS survey spectrum of $MnPS_3$.

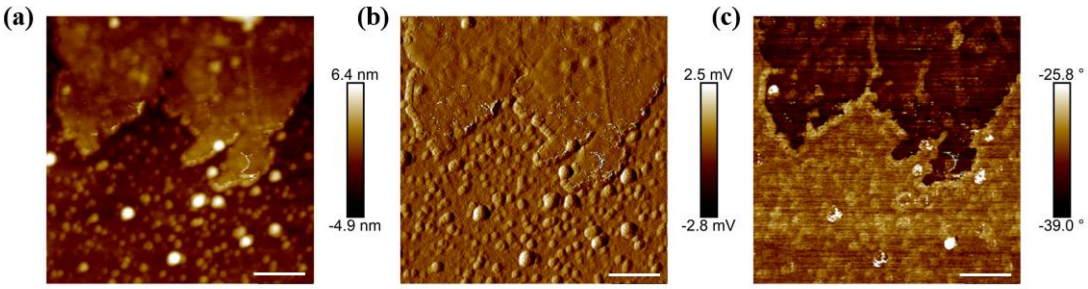

Figure S3. (a) PFM topography image, (b) amplitude image, (c) phase image of $MnPS_3$.

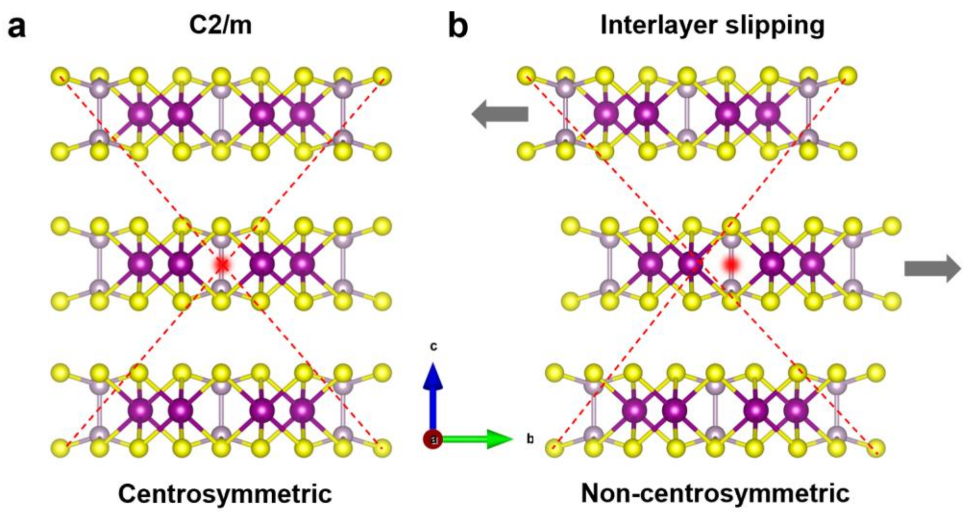

Figure S4. Crystal structures of 2D $MnPS_3$ at (a) non-slipped and (b) slipped regions.

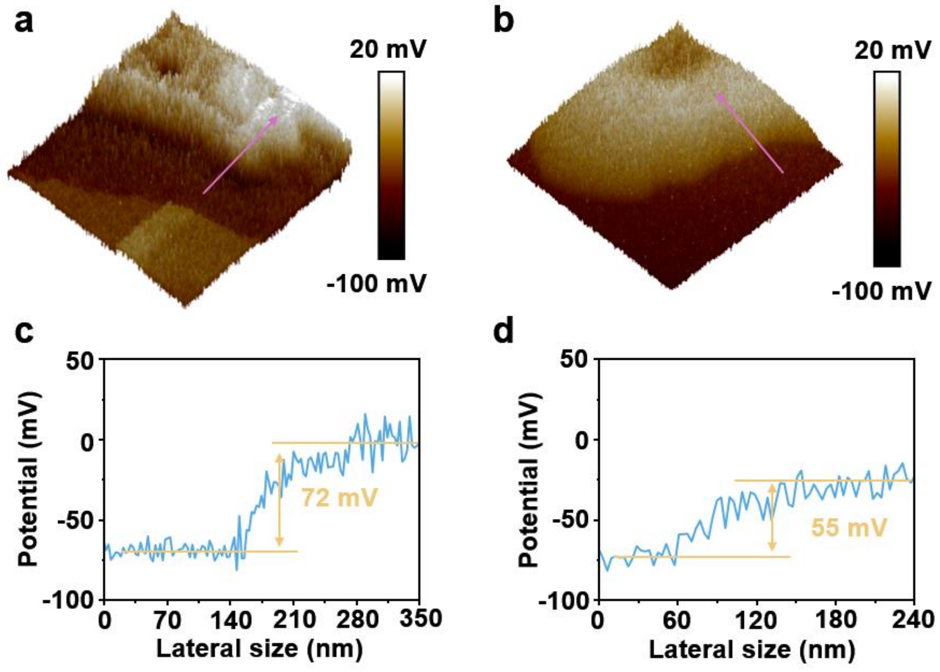

Figure S5. KPFM potential maps (a, b) and corresponding potential distribution profiles (c, d) of $MnPS_3$ NSs (a, c) and $MnPS_3$ NSs under mechanical stress (b, d).

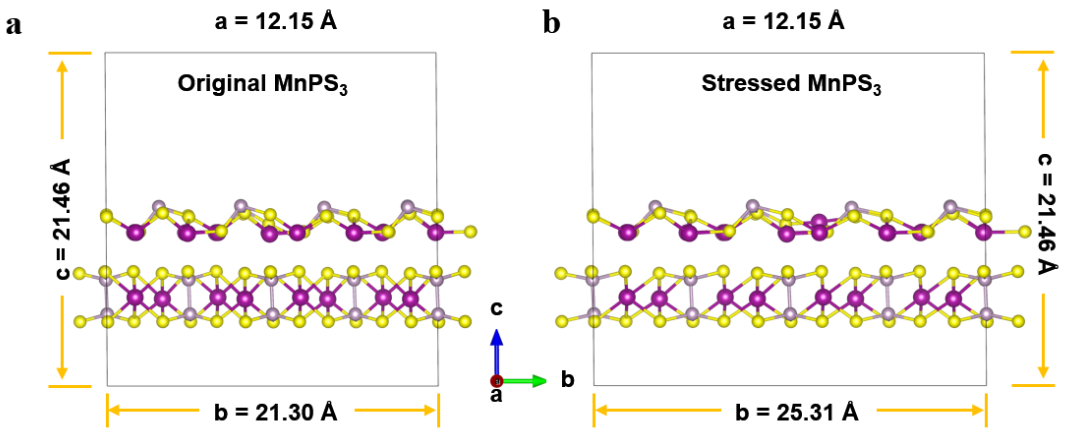

Figure S6. Optimized structure of (a) original $MnPS_3$ and (b) stressed $MnPS_3$.

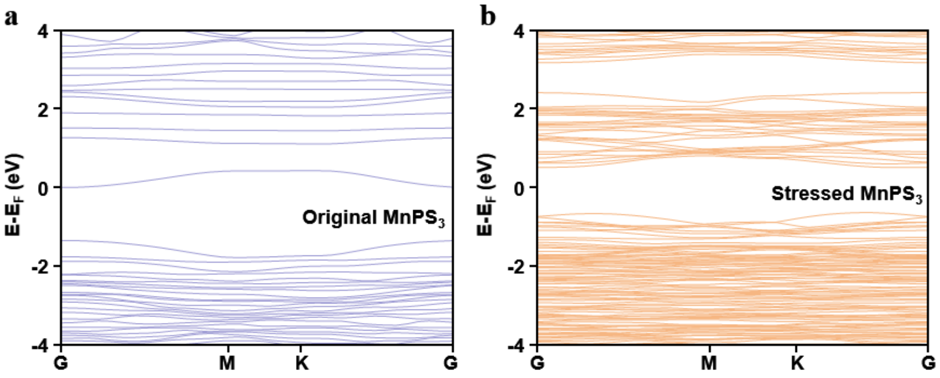

Figure S7. Calculated band structure of (a) original $MnPS_3$ and (b) stressed $MnPS_3$.

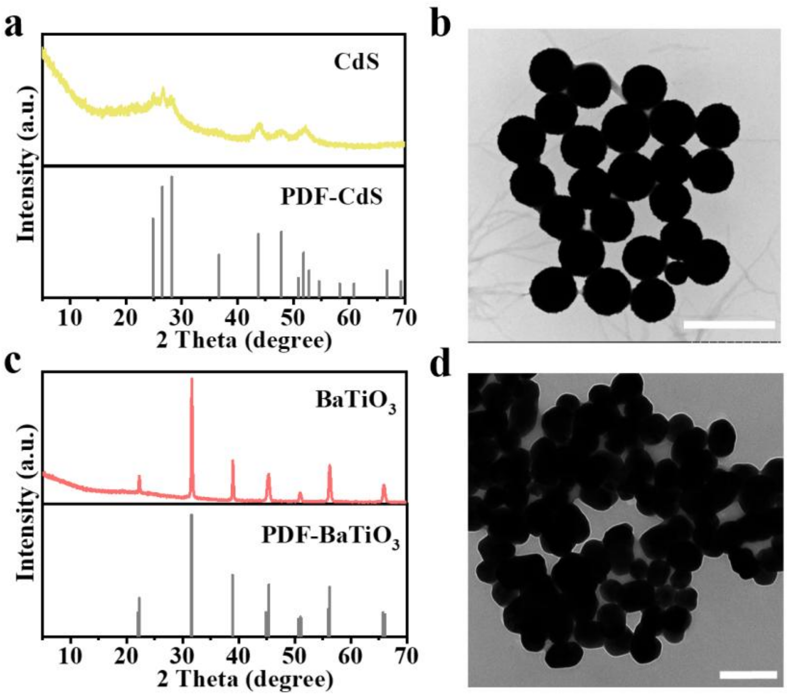

Figure S8. XRD patterns of CdS (a) and (c) $BaTiO_3$, TEM images of CdS (b) and (d) $BaTiO_3$.

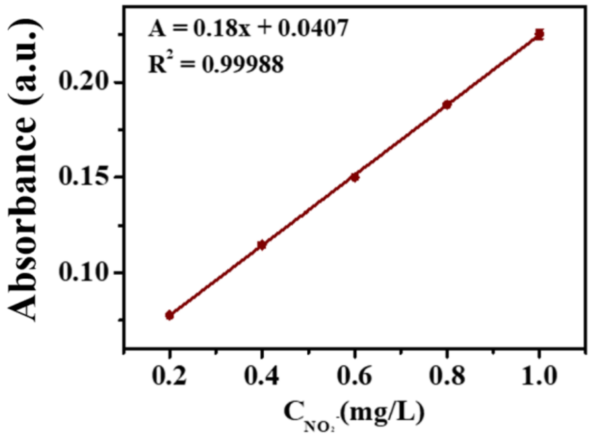

Figure S9. Concentration-absorbance calibration curve of NO_2^-.

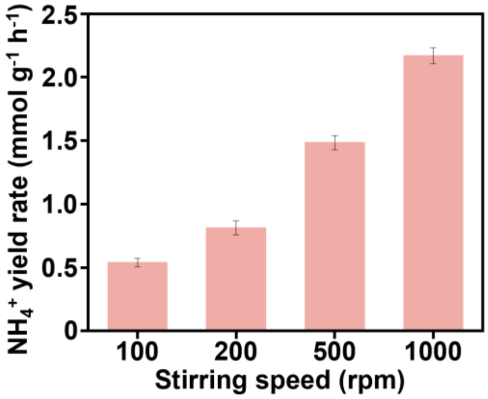

Figure S10. NH_4^+ yield rates under different stirring speeds in simulated seawater.

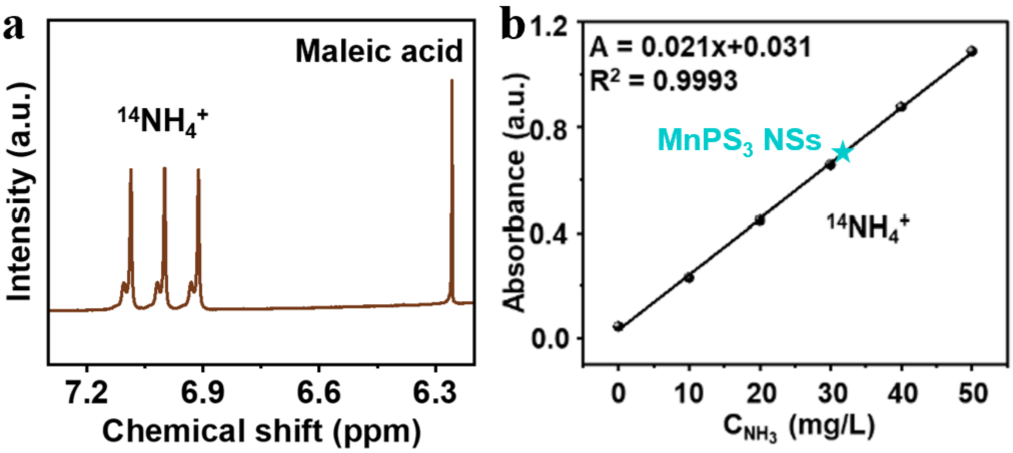

Figure S11. (a) ^{1}H NMR spectrum of $^{14}NH_4^+$. (b) Concentration-absorbance calibration curve of $^{14}NH_4^+$.

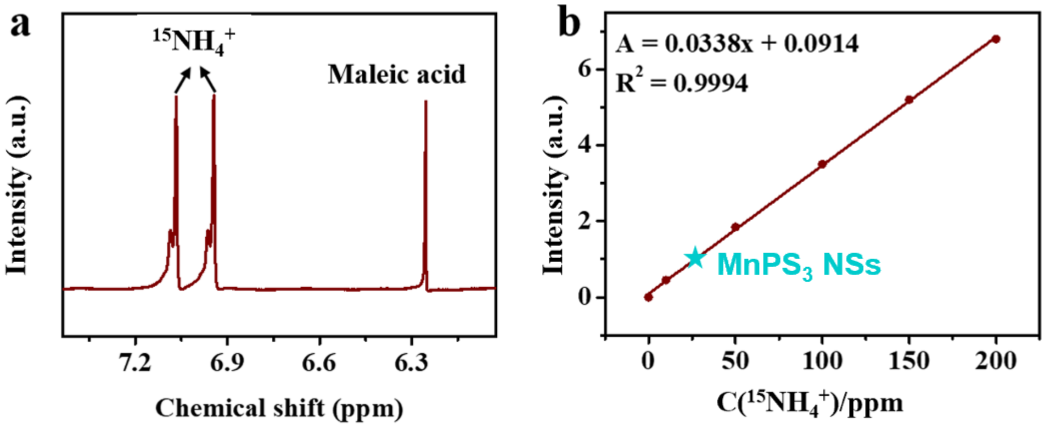

Figure S12. (a) ^1H NMR spectrum of $^{15}NH_4^+$. (b) Concentration-absorbance calibration curve of $^{15}NH_4^+$.

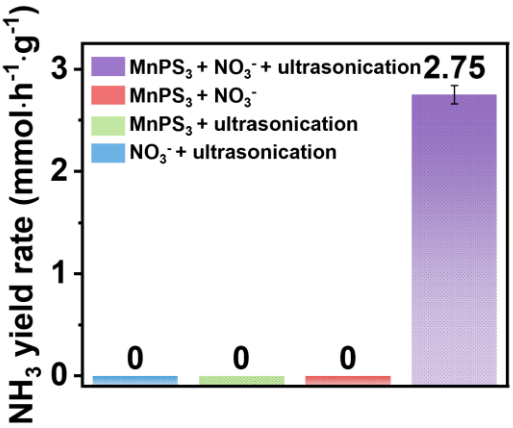

Figure S13. NH_4^+ yield rate at different reaction conditions.

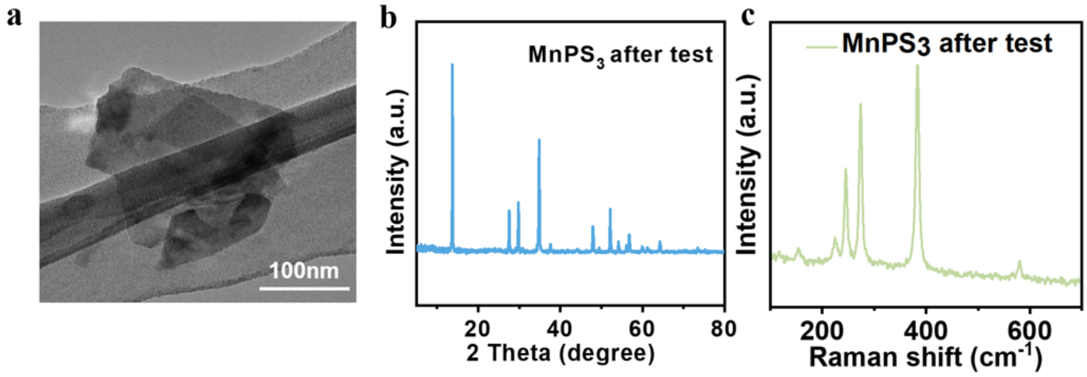

Figure S14. (a) TEM image, (b) XRD pattern, (c) Raman spectrum of $MnPS_3$ nanosheet after reaction.

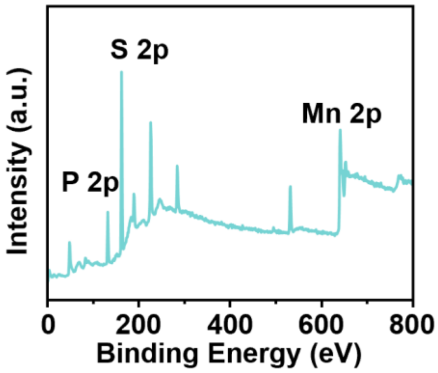

Figure S15. XPS survey spectrum of $MnPS_3$ NSs after reaction.

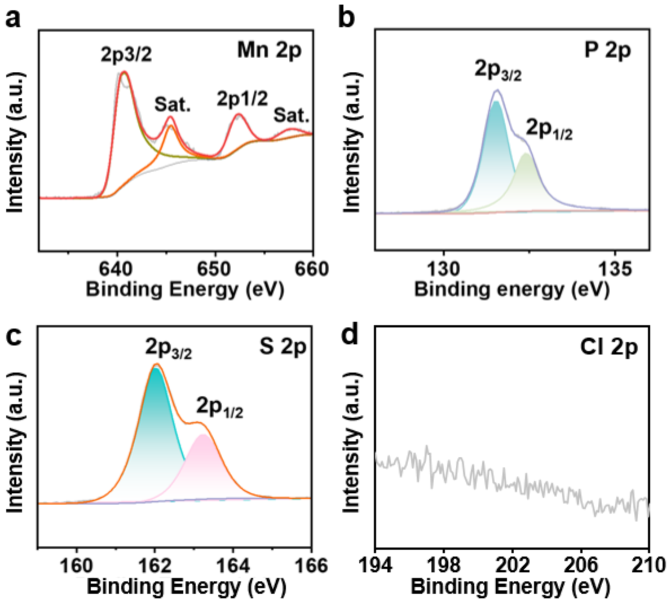

Figure S16. High-resolution XPS spectra of (a) Mn 2p; (b) P 2p; (c) S 2p (d) Cl 2p of $MnPS_3$ nanosheet after reaction.

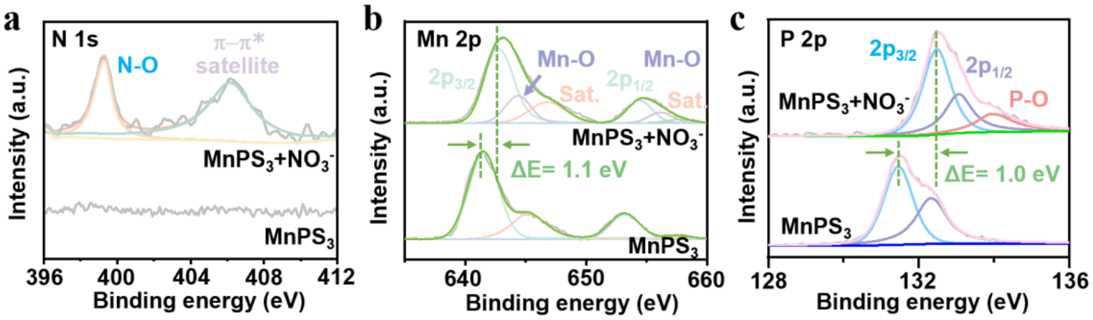

Figure S17. High-resolution XPS spectra of (a) N 1s, (b) Mn 2p and (c) P 2p of $MnPS_3$ NSs and

$MnPS_3$-NO_3^-.

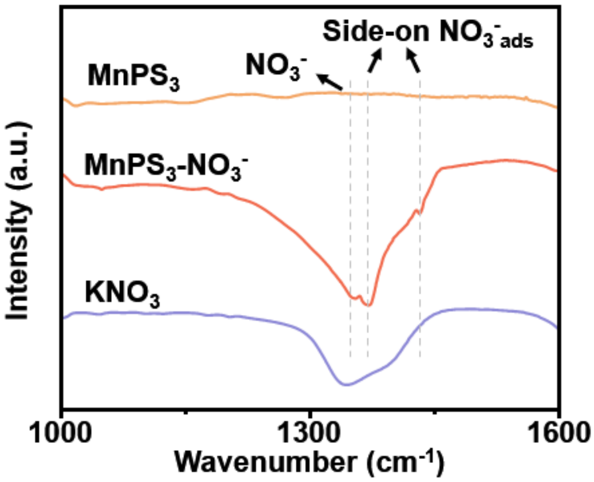

Figure S18. DRIFTS spectra of $MnPS_3$, $MnPS_3$-NO_3^- and KNO_3.

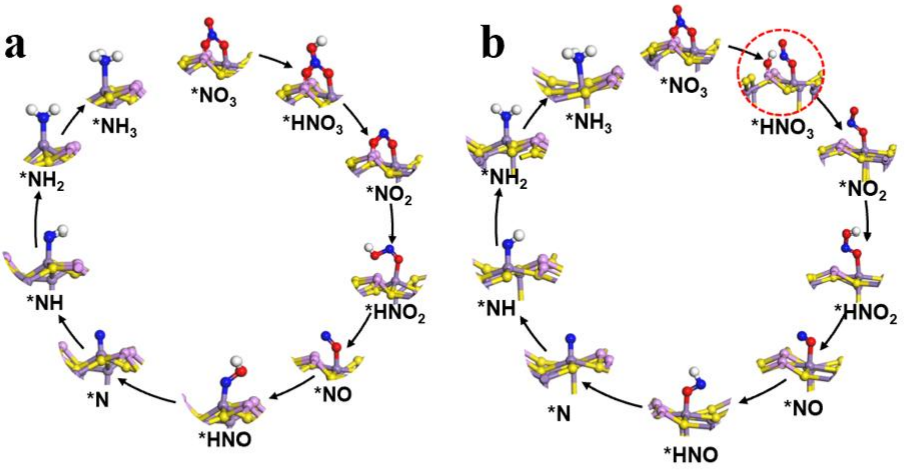

Figure S19. Different adsorption configurations of each step in NitRR.

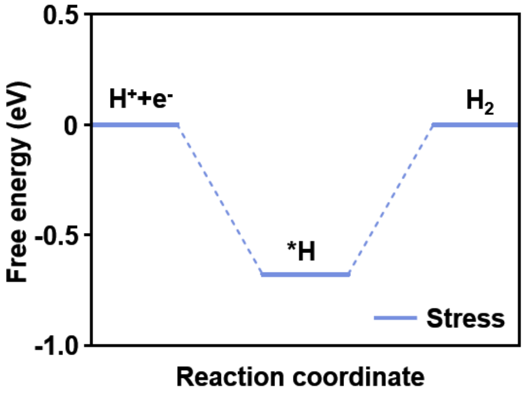

Figure S20. Free energy diagram of the *H adsorption and coupling on stretched $MnPS_3$ NSs.

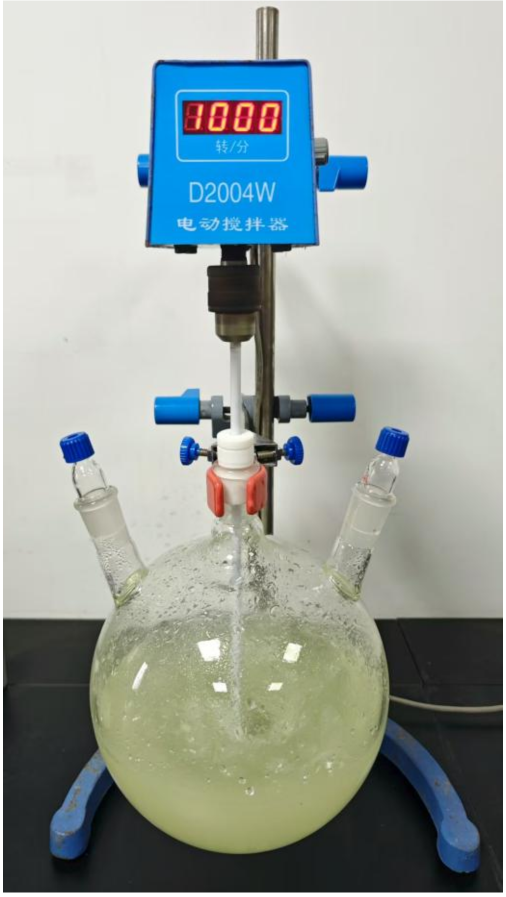

Figure S21. Photograph of piezocatalytic NitRR set-up in real seawater under stirring conditions.

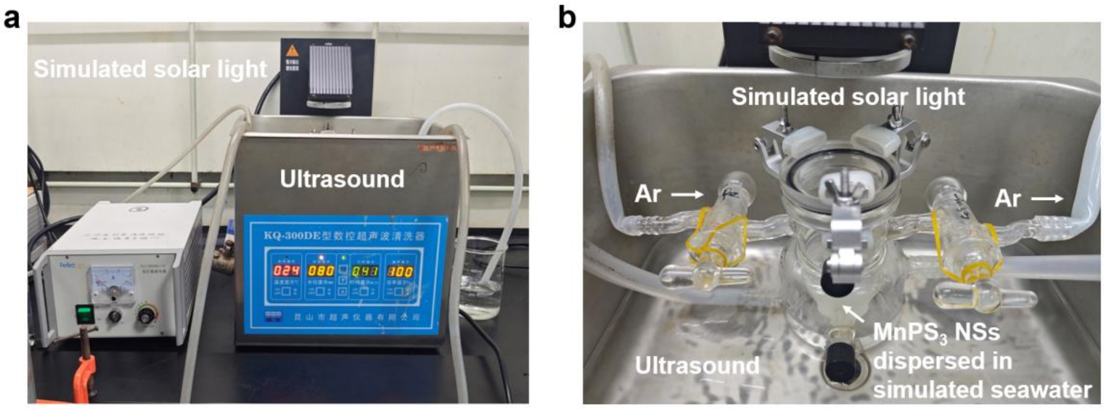

Figure S22. Photograph of piezocatalytic NitRR set-up in simulation seawater.

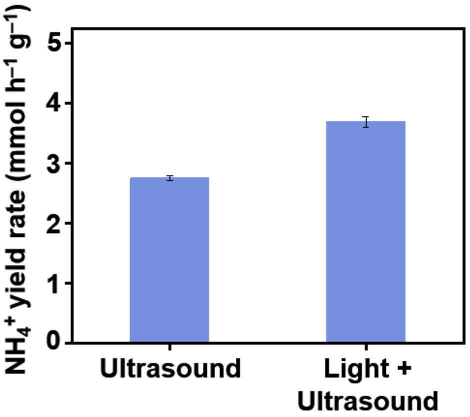

Figure S23. NH_4^+ yields of $MnPS_3$ NSs under ultrasound and solar-assisted ultrasound conditions in simulated seawater.

Table S1. Comparison of NH_3 production performance with references

Catalysts	Solution and raw material	NH_4^+ yield rate (mmol h^{-1} g^{-1})	Reference
CdS/Bi_2WO_6	N_2	0.1	[35]
ZnO-OV	N_2	0.01	[36]
Bi_5O_7Br	0.1 M KNO_3	1.38	[37]
Cu_2O@CuO/CNNTs	0.4 mM NO_3^-	1.38	[38]
NVs-g-C_3N_4	100 mg-N L^{-1}	1.24	[39]
CoS_x/ZnS	50 ppm	1.175	[40]
Ni/H_xWO_{3-y}	200 μmol KNO_3	1.05	[41]
1T-MoS_2/CdS	50 μmol KNO_3	0.78	[42]
Ag_2O/P25	100 μmol KNO_3	0.732	[43]
[NiFe]S	100 μmol KNO_3	0.58	[44]
$Bi/Bi_2Sn_2O_7$	0.4 mM KNO_3	0.2849	[45]
$Ta_3N_5/CdIn_2S_4$	0.4 mM KNO_3	0.2563	[46]

MIL-125(Ti)-250	100 mg L^{-1} KNO_3	0.1569	[47]

Table S2. Atomic ratio of $MnPS_3$ NSs before and after catalytic test.

Catalyst	Atomic ratio of Mn/P/S
$MnPS_3$ before test	1:0.98:2.97
$MnPS_3$ after test	1:0.98:2.95

References

1. Ma J, Jing S, Wang Y et al. Piezo-Electrocatalysis for CO_2 Reduction Driven by Vibration. Adv. Energy Mater. 2022; 12(27): 2200253.

2. Yuan J, Feng W, Zhang Y et al. Unraveling Synergistic Effect of Defects and Piezoelectric Field in Breakthrough Piezo-Photocatalytic N_2 Reduction. Adv. Mater. 2024; 36(5): 2303845.

3. G. Kresse, J. Furthmuller, Efficiency of ab-initio total energy calculations for metals and semiconductors using a plane-wave basis set, Computational Materials Science, 6 (1996) 15-50.

4. G. Kresse, J. Furthmuller, Efficient iterative schemes for ab initio total-energy calculations using a plane-wave basis set, Physical Review B, 54 (1996) 11169-11186.

5. J.P. Perdew, K. Burke, M. Ernzerhof, Generalized gradient approximation made simple (vol 77, pg 3865, 1996), Physical Review Letters, 78 (1997) 1396-1396.

6. G. Kresse, D. Joubert, From ultrasoft pseudopotentials to the projector augmented-wave method, Physical Review B, 59 (1999) 1758-177
